# Structural Elucidation of Agrochemical Metabolic Transformation Products Based on Infrared Ion Spectroscopy to Improve In Silico Toxicity Assessment

**DOI:** 10.1021/acs.chemrestox.3c00316

**Published:** 2023-12-20

**Authors:** Matthias J. A. Vink, Jimmy Alarcan, Jonathan Martens, Wybren Jan Buma, Albert Braeuning, Giel Berden, Jos Oomens

**Affiliations:** † Institute for Molecules and Materials, FELIX Laboratory, Radboud University, Toernooiveld 7, 6525 ED Nijmegen, The Netherlands; ‡ Department of Food Safety, German Federal Institute for Risk Assessment, Max-Dohrn-Str. 8-10, 10589 Berlin, Germany; § van’t Hoff Institute for Molecular Sciences, University of Amsterdam, Science Park 904, 1098 XH Amsterdam, The Netherlands

## Abstract

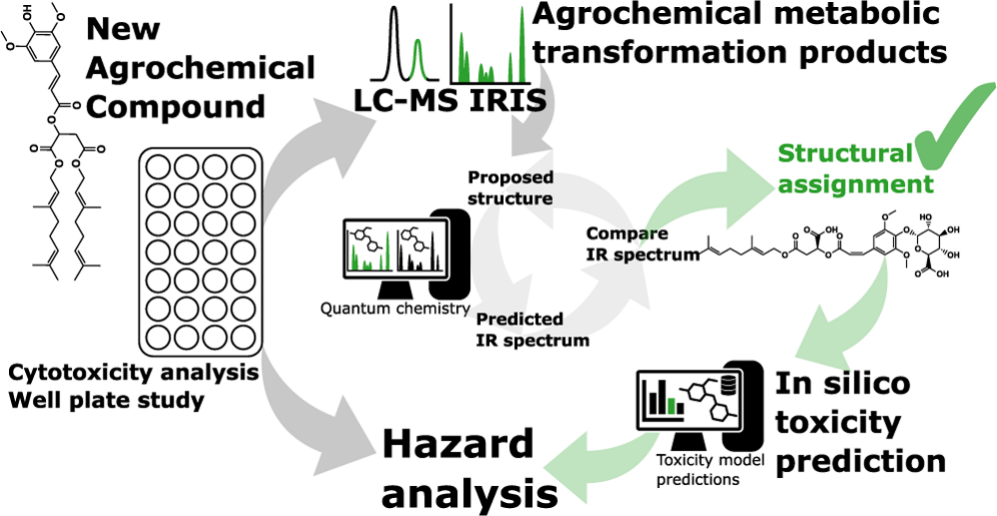

Toxicological assessments of newly developed agrochemical agents consider chemical modifications and their metabolic and biotransformation products. To carry out an in silico hazard assessment, understanding the type of chemical modification and its location on the original compound can greatly enhance the reliability of the evaluation. Here, we present and apply a method based on liquid chromatography–mass spectrometry (LC–MS) enhanced with infrared ion spectroscopy (IRIS) to better delineate the molecular structures of transformation products before in silico toxicology evaluation. IRIS facilitates the recording of IR spectra directly in the mass spectrometer for features selected by retention time and mass-to-charge ratio. By utilizing quantum-chemically predicted IR spectra for candidate molecular structures, one can either derive the actual structure or significantly reduce the number of (isomeric) candidate structures. This approach can assist in making informed decisions. We apply this method to a plant growth stimulant, digeraniol sinapoyl malate (DGSM), that is currently under development. Incubation of the compound in Caco-2 and HepaRG cell lines in multiwell plates and analysis by LC–MS reveals oxidation, glucuronidation, and sulfonation metabolic products, whose structures were elucidated by IRIS and used as input for an in silico toxicology assessment. The toxicity of isomeric metabolites predicted by in silico tools was also assessed, which revealed that assigning the right metabolite structure is an important step in the overall toxicity assessment of the agrochemical. We believe this identification approach can be advantageous when specific isomers are significantly more hazardous than others and can help better understand metabolic pathways.

## 1 Introduction

The potential impact of agrochemicals on ecosystems and human safety is an issue of continuous concern. The assessment of their toxicity through studies in the laboratory (in vitro), on living organisms (in vivo), and more recently with computational tools (in silico) is therefore essential.^1−3^ Metabolic transformation of agrochemicals can generate additional byproducts, possibly with higher toxicity.^4−7^ Full structural identification of these metabolites provides the necessary information to evaluate their toxicity and may also hold the key to designing better and safer agrochemicals.

Liquid chromatography–mass spectrometry (LC–MS) is a valuable tool for detecting and quantifying metabolites,^8−11^ although identifying compounds in terms of their full molecular structure can be challenging, particularly when dealing with multiple possible isomers. The analytical chemistry toolbox offers various methods to elucidate the chemical structure of metabolites, including tandem mass spectrometry, nuclear magnetic resonance (NMR) spectroscopy, and radiolabeled analysis of samples.^8−20^ Tandem mass spectrometry (MS/MS) is sensitive and fast, but data interpretation relies strongly on external standards collected in various libraries. On the other hand, NMR spectroscopy can resolve chemical structures without database references,^8−11,21−25^ but extensive purification is usually required, which is challenging, especially in multiwell plate in vitro studies.^12,13,20,23−27^ Much progress has recently been achieved using infrared ion spectroscopy (IRIS), where IR spectra of mass-to-charge selected species in an LC–MS workflow are measured.^28−37^ IRIS combines the sensitivity and selectivity of MS with the structural diagnostics of IR spectroscopy and has been successfully applied to identify, for instance, drug and plant metabolites.^28−34,36,38−40^

**Figure 1:**
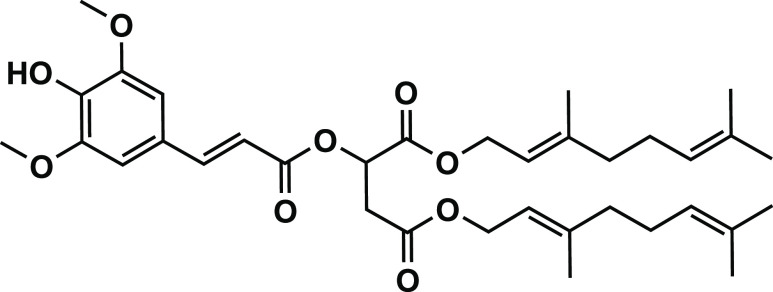
Chemical structure of digeraniol sinapoyl malate.

In this study, we use IRIS to characterize metabolites produced in human cells exposed to agrochemicals. We focus on a specific agrochemical designed to convert (harmful) ultraviolet solar light into heat. This so-called photon-to-molecule heater agrochemical is used as a foliar spray to enhance crop productivity, potentially extending the growing season without requiring the use of a greenhouse.^41−50^ In particular, we examine digeraniol sinapoyl malate (DGSM, see Figure 1), derived from plants’ naturally occurring sinapoyl malate. Synaptic acids can effectively filter UV radiation by absorption into various low-lying, electronically excited states. Rapid internal conversion to the ground electronic state then releases the energy as heat. The part of the solar spectrum not used for photosynthesis can thus be employed to raise crop temperatures and boost crop yields.^47,51−53^ Facile excited-state cis–trans isomerization drives the fast internal conversion, effectively providing efficient UV-to-heat conversion pathways.^54,55^ DGSM has two lipophilic tails that enable the molecule to stick to the wax layer of leaves, making it resistant to rainwater wash-off. However, this feature also raises concerns regarding intake through consumption. Thus, it is vital to analyze and characterize the metabolic processing of DGSM to protect against potential hazards and the toxicity of its metabolic transformation products.

Selecting a relevant cell line to provide a suitable human in vitro model is essential for assessing the fate and safety of the studied compounds. Using human-specific cell lines gives the advantage of producing human-specific metabolites compared to rodent models that may produce different metabolites (i.e., interspecies differences). For this aim, we chose the Caco-2 cell line for its widespread use as a model of the intestinal epithelial barrier relevant to the intake of compounds in the food chain.^56^ Additionally, we chose the HepaRG cell line as it has the potential to be a substitute for primary human hepatocytes and is particularly suitable as an in vitro model in drug metabolism and disposition studies, as well as multiwell plate toxicity studies.^57,58^ Although these cell lines may be used to monitor a variety of end points, for example, cell death, they do not inherently provide insight into the chemical modification of the test compounds, as they do not inherently elucidate the chemical structure but rather the effect of these modifications. Nevertheless, knowledge of disposal mechanisms and transformation products is crucial for improving the design of safer agrochemicals.

To elucidate the chemical structure of metabolites produced in the cell lines, we used IRIS in an LC–MS workflow to obtain IR spectra of the metabolites in combination with a quantum-chemistry workflow for obtaining reference spectra. In silico toxicity prediction tools then assess the identified products to estimate their potential hazards. In silico quantitative structure–activity relationship (QSAR) predictions are commonly used for safety evaluations by virtue of their cost-effectiveness. However, these in silico platforms rely on an accurate chemical structure for assessment, which is trivial for the agrochemical itself but not for its transformation products. In order to ensure reliable results from in silico platforms, it is necessary to have an accurate chemical structure for evaluation. When isomeric structures cannot be differentiated, it is typically assumed that the most hazardous isomer is present, which is the safest conclusion but likely incorrect, especially when many potential isomers are included in the toxicity screening. This can undermine confidence in the assessment. Here, we demonstrate how IRIS can aid in establishing the required molecular structure or at least eliminate many potential isomeric structures.

## 2 Methods

### 2.1 Chemicals

Prof. Florent Allais (URD ABI Agroparistech) kindly provided the DGSM. Dimethyl sulfoxide (DMSO) was purchased from AppliChem (Darmstadt, Germany). All other chemicals were purchased from Merck (Darmstadt, Germany) or Sigma Aldrich at LC–MS grade or higher.

### 2.2 In Vitro Metabolism Investigation: Cell Culture

Human Caco-2 colorectal adenocarcinoma cells were obtained from the European Collection of Cell Cultures (Salisbury, UK) and cultured as described in detail by Voss et al.^59^ In brief, cells (passage 26–36) were seeded at 10,000 cells/well in 96-well plates in appropriate culture medium (DMEM with 4.5 g/L glucose, l-glutamine, sodium pyruvate, and 3.7 g/L NaHCO_3_) supplemented with 10% heat-inactivated fetal bovine serum, 100 IU/mL penicillin, and 100 μg/mL streptomycin. Cells were maintained at 37 °C in a humidified atmosphere containing 5% CO_2_. For differentiation into an intestinal epithelial-like monolayer, cells were cultured for 3 weeks with medium renewal every 2–3 days.^60^

Human HepaRG hepatocarcinoma cells (Biopredic International, Saint-Grégoire, France) were cultured as previously described in Alarcan et al.^61^ Briefly, cells (passage 15–20) were seeded at 9000 cells/well in 96-well plates in appropriate culture medium (Williams’ E Medium with stable glutamine, 2.24 g/L NaHCO_3,_ and phenol red) supplemented with 10% heat-inactivated fetal bovine serum, 100 IU/mL penicillin, 100 μg/mL streptomycin, 5 μg/mL insulin, and 50 μM hydrocortisone hemisuccinate. Cells were maintained at 37 °C in a humidified atmosphere containing 5% CO_2_. After 2 weeks of cultivation with culture medium, the cells were cultured for 2 weeks in the same medium supplemented with 1.7% DMSO (differentiation medium). During 4 weeks of cultivation, the medium was renewed every 2 to 3 days.

### 2.3 In Vitro Metabolism Investigation: Cell Viability Assay

The cytotoxicity of DGSM in Caco-2 and HepaRG cells was evaluated by using the neutral red uptake (NRU) assay. After 24 h of treatment, cell supernatants were collected and stored at −81 °C until further analysis, while cells were incubated with 100 μL of neutral red solution (4 μg/mL) for 2 h. After being washed with phosphate-buffered saline (PBS), cells were lysed and put under shaking for 10 min before fluorescence measurement at 645 nm (excitation at 530 nm).

### 2.4 Metabolites Investigation

The cell viability assay samples were transferred from the multiwell plates to 2 mL Eppendorf vials and transported on dry ice from the BfR institute to the FELIX Laboratory. The vials were stored at −20 °C until analysis. Before analysis, the Eppendorf vials were allowed to defrost for approximately 1 h at 5 °C in the refrigerator. After the vial contents were defrosted, they were transferred to an LC analysis vial. The Bruker Elute HPLC system was utilized, featuring a column oven and an autosampler coupled to a Bruker AmaZon ion trap mass spectrometer. For high-resolution accurate mass (HRAM) analysis, the system was instead coupled to a Bruker SolariX Fourier-transform ion cyclotron resonance (FTICR) MS. The autosampler was maintained at 4 °C during separation, while the column oven was kept at 40 °C. Separation was performed on a Waters Acquity UPLC HSS T3 reversed-phase C18 column with dimensions of 2.1 × 150 mm, 1.8 μm particle size, and 100 Å pore size. Injections of 2 μL were made. A linear gradient from 10% solvent A (0.1% FA in water) and 90% solvent B (0.1% FA in MeCN) was used for elution at a flow rate of 0.4 mL/min flow rate. After 3 min, this was changed to 100% solvent B in 1 min. The conditions were held for one min before being reverted to the initial conditions in one min and kept for another 2 min to allow for column equilibration. We chose this (very) short method to maintain relevance to a high-throughput screening setting for metabolomics.

The Bruker AmaZon ion trap was connected to the LC and equipped with a two-position, six-port divert valve for fractionation.^28,29,37^ The elution time of the metabolite of interest was determined using a mass spectrometer to fractionate it from the biological matrix. Five injections were fractionated by programming the divert valve to divert the flow to a sample vial at the observed elution time. The acquired fractionated sample was diluted by approximately 1:1 in MeCN before direct infusion ESI utilizing a Hamilton 250 μL syringe.^30,32^

### 2.5 IRIS Characterization

IRIS was performed by using the tunable IR radiation from the FELIX free-electron laser. Experiments were carried out in a modified Bruker AmaZon quadrupole ion trap (QIT) mass spectrometer, providing optical access to the trapped ion population. Hardware and software modifications allow one to synchronize the ion trap sequence with the FELIX IR pulse train, as described in detail previously.^62,63^

The ion of interest is mass-isolated in the QIT and irradiated with a single macro pulse from FELIX, after which a fragmentation mass spectrum is recorded. Upon resonance of the laser frequency with a vibrational transition in the ion, the mass spectrum displays the IR-induced fragment ions as well as the remaining precursor ion. By recording a sequence of mass spectra while tuning the laser frequency, we can construct an IR ion spectrum from the IR-induced fragment yield of the various wavelengths.

After irradiation, the IR dissociation yield can be calculated from the precursor (*I*_P_) and fragment (∑*I*_F_) ion intensities. The yield, defined in eq 1, is directly proportional to the ions’ dissociation rate and can thus be interpreted as the vibrational absorption spectrum of the ions.^63^
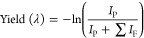
1

The laser frequency is scanned over the 550–3700 cm^–1^ range in either 3 or 5 cm^–1^ increments. Per wavelength step, 4 to 8 mass spectra are averaged.^63^ A grating spectrometer is employed for wavelength calibration, while laser power is measured to allow for frequency-dependent calibration of pulse energy, which is used to correct the IR dissociation yield linearly.^63^

This experimental IR spectrum can be matched against reference IRIS spectra measured from physical standards or against quantum-chemically computed IR spectra^30,62,64−70^ obtained through a workflow described below. Chemical structures used as input for the computations are derived from chemical intuition aided by the accurate mass, MS/MS fragments, the metabolite’s retention shift, and its IR ion spectrum, which can indicate the absence or presence of specific functional groups. Additionally, we can use in silico tools, such as SOMP^71^ and GLORYx,^72^ to predict the reactivity of specific groups or possible metabolic products. The SOMP tool predicts the sites that are likely to interact with certain enzymes responsible for phase II metabolism. On the other hand, GLORYx provides a list of predicted metabolites that may be formed through phase I and phase II metabolism in humans, ranked by the likeliness of certain sites to be involved in the metabolic transformation.

### 2.6 Cheminformatics Workflow for Computation of IR Spectra

Our computational workflow uses the cheminformatics toolbox RDKit in Python 3 and the Gaussian16 quantum chemistry software suite. For an input structure, we assume that all oxygen and nitrogen atoms can be (de)protonation sites or coordinate with Na^+^ ions.^29,30,72,73^ 500 random 3D conformations are then generated and minimized using the MMFF94 force field. The random conformation set is optimized at the semiempirical PM6 level, followed by vibrational analysis.^74^ Duplicates and structures with broken bonds are removed, and a relative energy cutoff of 40 kJ/mol, determined by the Gibbs free energies using PM6, is used to filter out unfavorable structures and (de)protonation sites. A limit of 20 conformers per original ionization site is set for optimization and harmonic frequency calculation at the DFT level (B3LYP/6-31++G(d,p)). Finding multiple low-energy conformations that may coexist in the ion population is not unusual. A Boltzmann-weighted average at 298.15 K is used to account for their contributions to the IR spectrum. In the fingerprint region, the harmonic IR frequencies were adjusted with a scaling factor of 0.975, while a scaling factor of 0.955 was applied for frequencies above 2500 cm^–1^.^75^ Furthermore, the calculated stick spectra were broadened by a 20 cm^–1^ full width at half the maximum Gaussian function to aid in comparison with the naturally broadened experimental spectra. Additionally, all spectra were normalized in intensity to facilitate a comparison of experimental and computed spectra. The resulting IR spectra are subsequently empirically matched to the recorded IRIS spectra.

### 2.7 In Silico Mutagenicity and Carcinogenicity

Several software tools were used to assess the potential mutagenicity and carcinogenicity, i.e. the VEGA, TEST, and LAZAR platforms. Detailed information on how the models were used can be found in previous publications.^76,77^ To combine the predictions generated by the individual models, their outputs were converted to numeric values ranging between 0 and 1, with presumed nonmutagenicity/noncarcinogenicity spanning the range 0–0.50, while mutagenicity/carcinogenicity ranges from 0.51 to 1.

The arithmetic mean of the different prediction scores was calculated and plotted in a diagram, divided into three zones: a score >0.66 means a positive prediction (mutagenic/carcinogenic) with good reliability. In contrast, a score <0.33 is a negative prediction (nonmutagenic/noncarcinogenic) with good reliability. Scores between 0.33 and 0.5 are regarded as negative predictions (nonmutagenic/noncarcinogenic) with insufficient reliability, while scores between 0.5 and 0.66 are regarded as positive predictions (mutagenic/carcinogenic), again with insufficient reliability.

### 2.8 In Silico Endocrine Toxicity

Endocrine toxicity was assessed using the VEGA platform. Five different models were employed, with four models providing predictions on receptor-mediated effects [i.e., estrogen receptor-mediated effect (IRFMN/CERAPP)], androgen receptor-mediated effect (IRFMN/COMPARA), thyroid receptor alpha effect (NRMEA), and thyroid receptor beta effect (NRMEA), and one model providing predictions on receptor binding affinity [estrogen receptor relative binding affinity model (IRFMN)]. Each model provides a qualitative prediction (yes/no) alongside information about the reliability of the prediction (low, moderate, or high reliability).

### 2.9 In Silico Acute and Short-Term Toxicity

Acute and short-term toxicity was investigated using in silico models for oral LD_50_ and the no-observed adverse-effect level (NOAEL) from 90 day toxicity studies. The oral LD_50_ in rats was estimated using TEST, based on a data set comprising values from 7413 substances. The NOAEL was estimated by calculating the average value between the predictions from the modules NOAEL (IRFMN-CORAL) and NOAEL (CONCERT/Coral) provided within the VEGA platform.

## 3 Results and Discussion

### 3.1 In Vitro Metabolism and Cytotoxicity

**Figure 2:**
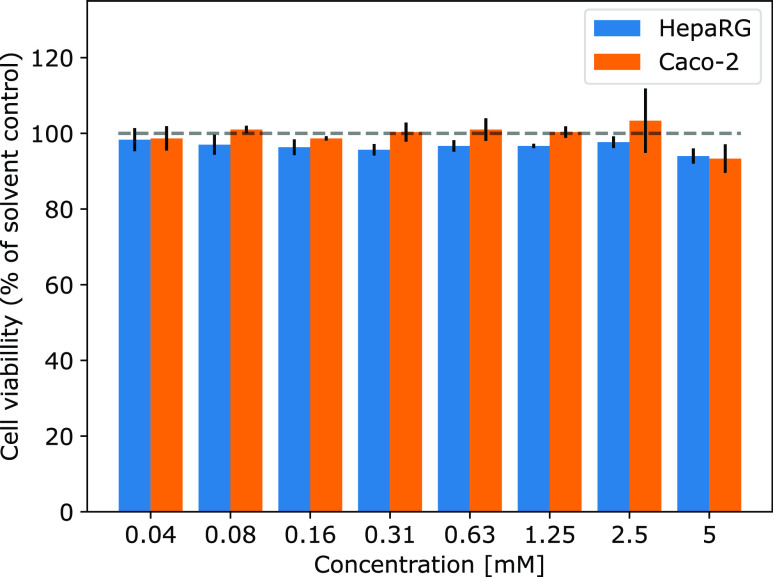
Cell viability in Caco-2 and HepaRG cells. Following 24 h of treatment with different concentrations of DGSM, cytotoxicity was measured using the NRU assay. Results were obtained from three independent experiments performed in triplicate (mean + SD).

Rat and/or human hepatic S9 fractions and liver microsomes are traditionally used for metabolism studies.^78^ Despite their evident usefulness, they do not mimic the true physiological situation due to their restricted spectrum of metabolic processes (e.g., microsomes can be used only for CYP and UGT reactions). Moreover, investigation into the toxicity of the produced metabolites is not directly possible, and their further incubation in a cellular model could be questionable as the metabolites, in that case, would not necessarily be taken up inside the cells. Thus, to cover the full range of metabolism processes (uptake, biotransformation, and efflux) and test for preliminary toxicity, we decided to use cellular models. Human primary hepatocytes are considered the most physiologically relevant model,^79,80^ but their availability is limited, and the existing interindividual differences of metabolic enzymes make it difficult to obtain consistent results.^80^ Therefore, we chose the human immortalized HepaRG cell line owing to its high expression of drug metabolism enzymes as well as transporters.^58,81^ We also used the human Caco-2 cells, as they can differentiate into enterocyte-like cells displaying tight junctions, microvilli on the apical side, and functional enzymes (e.g., phase II metabolism enzymes).^60,82^ After 24 h of incubation with DGSM, no cytotoxicity in Caco-2 or HepaRG cells was detected in the NRU assay for DGSM concentrations up to 5 mM, as can be deduced from Figure 2. Supernatants from cells treated with the highest concentration level were analyzed using LC–MS for metabolite detection, including the respective controls. The nature of the metabolic products may provide a better understanding of the metabolic pathways and allow us to assess the potential toxicity of the metabolic transformation products.

### 3.2 LC–MS Analysis of Metabolic Products

**Figure 3:**
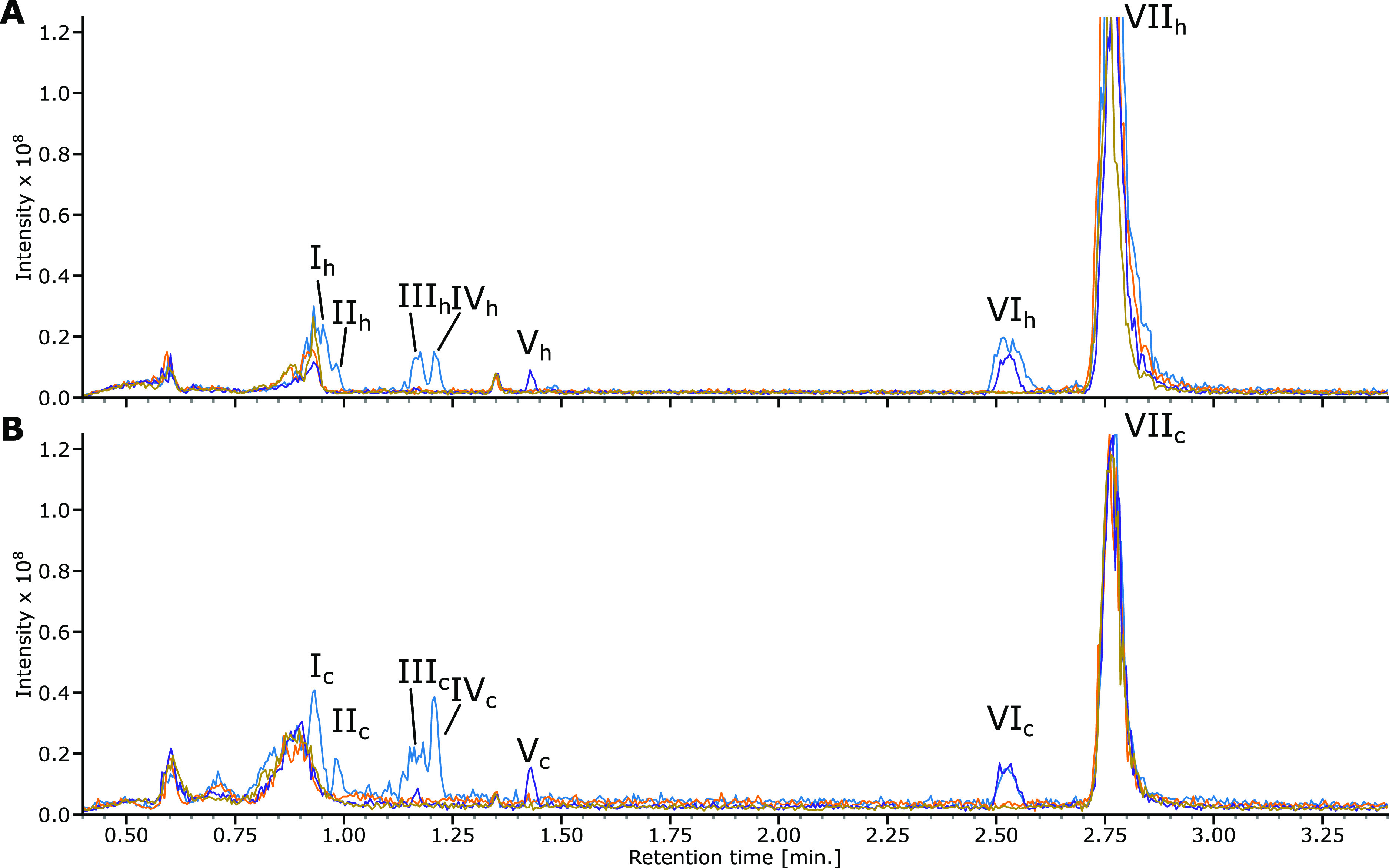
BPC curves of LC–MS analysis of cell line assays. (A) Caco-2 cell line assay extracts, (B) HepaRG cell line assay extracts. In both panels, the blue trace represents incubation with DGSM, the purple curve represents DGSM incubation without cells being present in the medium, the orange trace represents cells incubated with DMSO, and the brown curve represents DMSO incubation without cells in the medium.

The LC–MS base peak chromatograms (BPC) are depicted in Figure 3 for both the Caco-2 and the HepaRG cell lines. The blue trace represents DGSM incubated with cells, whereas the purple curve represents DGSM incubated only in the cell medium. This latter curve is the negative control for off-target chemical reactivity with the cell medium. The orange trace in both panels represents the solvent control, i.e., cells in which 1% DMSO was incubated, whereas the brown trace represents the solvent without cell incubation. These last two samples were used as negative controls to eliminate any products from the cells or their growth medium.

The blue traces in Figure 3 show that four chromatographic features (I, II, III, and IV) relate to the metabolization of DGSM in the cell media and that Caco-2 and HepaRG analyses have similar chromatographic features. Feature V relates to DGSM reacting with the growth medium in the absence of the cells, feature VI is DGSM itself, and peak VII relates to the cell medium itself. Peaks V–VII are thus not further investigated. In negative ion mode, we found an additional metabolite in the HepaRG cell line at a retention time of approximately 2.25 min, as depicted in Figure S1 of the Supporting Information.

When we probe which ions contribute to the chromatographic features, as shown in Table 1, we conclude that some *m*/*z* values appear more than once. Furthermore, we note that the labeled features and the underlying ions have identical MS/MS spectra, indicating that the same metabolites are formed in the Caco-2 and HepaRG cell lines, where some metabolites are formed as multiple isomers. Table 1 summarizes the results and displays the HRMS values and chemical formulas.

**Table 1:** Overview of Ions of Interest and Their Associated Chemical Formulas from Both Cell Line Characterizations^a^

metabolite short name	ion (*m*/*z*)	found in labelled	HRAM	suggested chemical formula	accurate mass	IRIS from
DGSM	635^(+)^	VI_C/H_		C_35_H_48_O_9_ (Na^+^)	635.319054	
MA	499^(+)^	III_C/H_, IV_C/H_	499.19597	C_25_H_32_O_9_ (Na^+^)	499.1939	IV_C_
MB	515^(+)^	I_C/H_, II_C/H_	515.19120	C_25_H_32_O_10_ (Na^+^)	515.1888	I_C_
MC	555^(−)^	SI_H_		C_25_H_32_O_12_S (−H)	555.1531	SI_H_
MD	675^(+)^	I_H_, II_H_	675.22950	C_31_H_40_O_15_ (Na^+^)	675.2260	II_H_

a DGSM and metabolites MA, MB, and MD are measured in positive mode, whereas MC is measured in negative mode.

To arrive at preliminary assignments for the DGSM metabolites of Table 1, we consider accurate masses, relative elution times, and likely metabolic relations to the parent molecule. Further, we use MS(/MS) interpretation to refine and substantiate the assignments.

**Figure 4:**
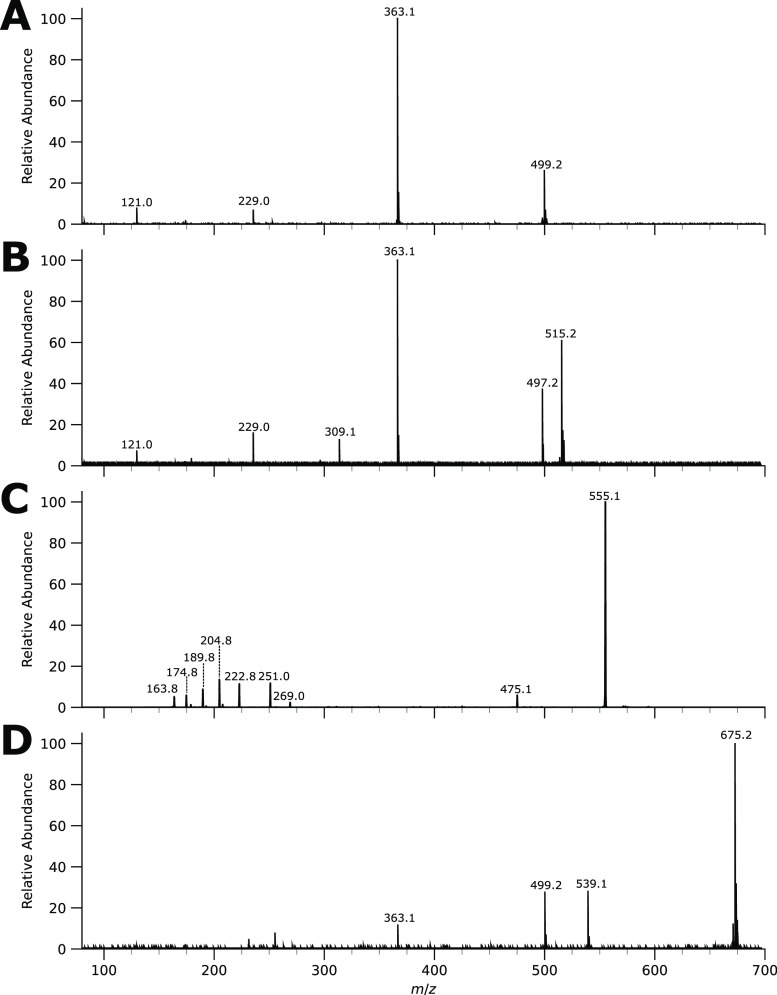
MS/MS spectra of MA, MB, MC, and MD (top to bottom). Note that the spectrum of MC was recorded on a QIT in the negative ion mode, whereas the others were recorded by employing an FTICR-MS for HRAM determination in the positive ion mode.

The HRAM mass of the *m*/*z* 499 metabolite (MA) suggests that it corresponds to ester cleavage, expelling one of the geranyl tails. The MS/MS spectrum in Figure 4 suggests that a second geranyl moiety is lost upon CID, forming the (sodiated) SM base structure at *m*/*z* 363. However, the MS/MS spectrum provides no information on which of the geranyl moieties is expelled. Hence, since the diganoiol tails are not symmetric, their ester cleavage may lead to two distinct isomeric forms of the *m*/*z* 499 product. Further, we will consider both *cis*- and *trans*-isomerized (upon which the UV-induced heating activity of DGSM is based)^29,41,47^ products for the *m*/*z* 499 ion, giving four possible isomers.

It appears that the *m*/*z* 499 ion (MA) is an intermediate metabolic product, as the accurate masses and corresponding molecular formulas of the other ions in Table 1 suggest further derivatization of this species. Specifically, the *m*/*z* 515 ion (metabolite MB) is likely due to hydroxylation of metabolite MA, which is supported by the MS/MS fragment at the *m*/*z* 497 ion (Figure 4B), characterized as a water loss typical for alcohols. Further, we note that the fragmentation pattern of MB is identical with that of MA, except for the *m*/*z* 309 fragment. This fragment is explained by a hydroxylated 4-(3,7-dimethylocta-2,6-dienoxy)-3-hydroxy-4-oxobutanoic acid, which is a hydroxylated ester-cleaved digeraniol tail of DGSM. The MS/MS spectrum contains no information on the exact site of hydroxylation but does suggest that it occurred on the geranyl tail.

The *m*/*z* 555 anion (MC) is attributed to the deprotonated sulfonation product of MA, based on the +79.96 Da increase in mass relative to [MA–H]^−^. When we examine its MS/MS spectrum depicted in Figure 4C, the *m*/*z* 475 ion can be explained as the deprotonated MA metabolite formed by the typical SO_3_ loss of sulfonates. The *m*/*z* 223 fragment is attributed to deprotonated sinapic acid, further confirming the structure of MC.

Finally, *m*/*z* 675 likely corresponds to the sodiated ion of the product of glucuronidation of metabolite MA (+176.03 Da), which is supported by the MS/MS fragmentation pattern in Figure 4D. The *m*/*z* 539 fragment corresponds to the expulsion of the geranyl tail, where the glucuronide remains attached to the metabolite, whereas the *m*/z 499 fragment corresponds to expulsion of the glucuronide, giving the sodiated MA metabolite.

**Figure 5:**
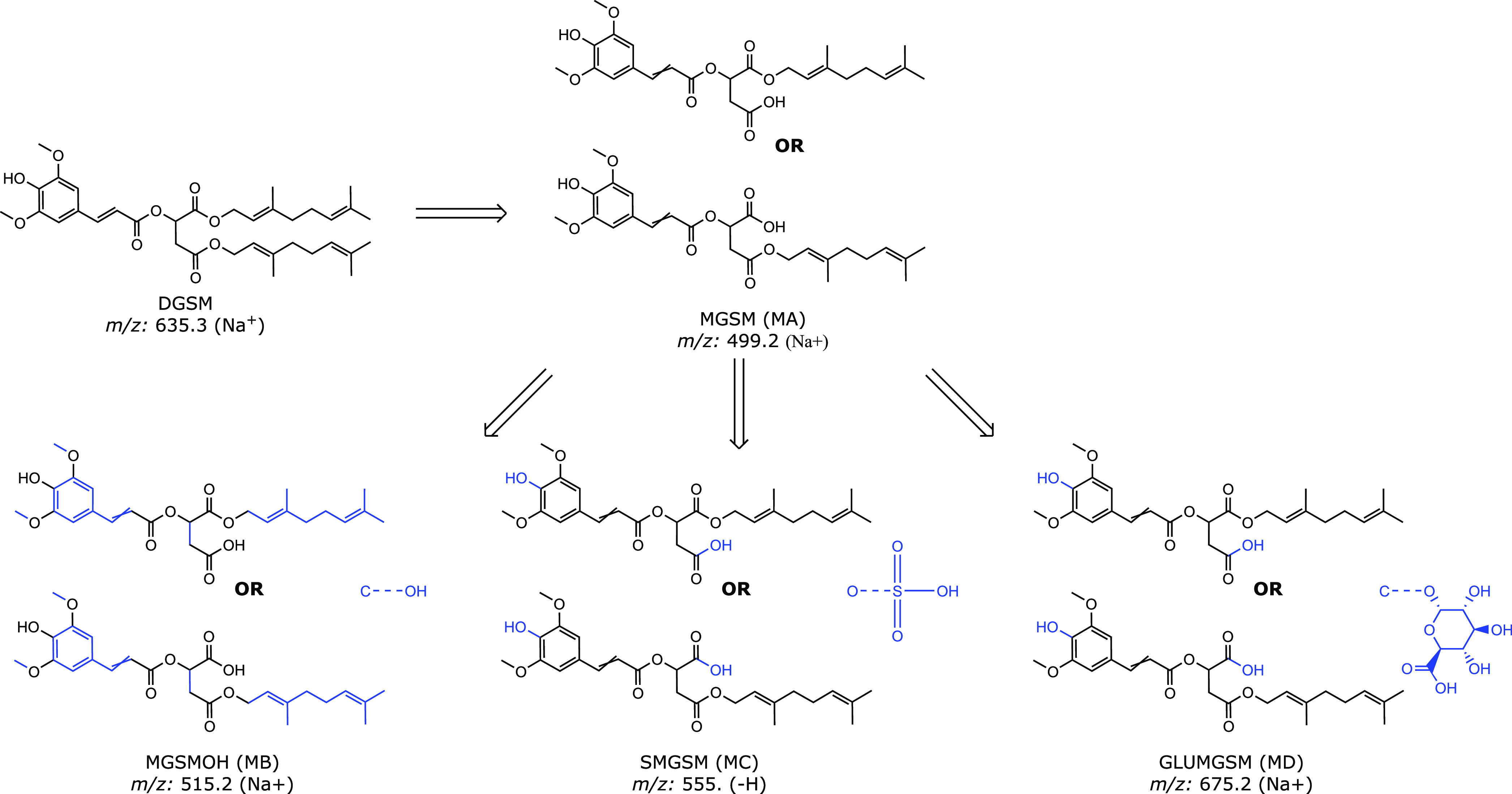
Proposed pathway of DGSM metabolization based on LC–MS characteristics. Moieties colored blue indicate likely transformation sites.

These metabolic modifications of DGSM are typical for phase I and II metabolism, and the metabolite structures derived from the MS/MS spectra are summarized in Figure 5. The proposed structures are further supported by their relative elution shift compared to DGSM, based on their estimated change in relative hydrophobicity.

Regarding possible isomeric structures of these metabolites, we begin with the metabolite MD, which could form from any of the four isomers of MA. Based on the literature, we know that the hydroxyl groups in MA are likely to undergo glucuronidation.^83−85^ However, since MA has three unique hydroxyl groups that can form diastereomers during glucuronidation, there are 32 possible stereoisomers of this species, of which 16 can be distinguished by IR spectroscopy. The presence of all of these isomers is unlikely, as we only find two chromatographic features containing the *m*/*z* 675 ion. The same arguments apply to the metabolite MC. Structure elucidation is more complex for metabolite MB as all carbons can undergo hydroxylation.

We can use in silico tools such as GLORYx^72^ and SOMP^71^ to predict which isomers are most likely for phase I and II metabolization products based on reactivity and enzymatic interaction. The results are depicted in Tables S1 and S2. In this analysis, we will consider DGSM and MGSM (MA) because of their central role in the proposed metabolization pathway (Figure 5). Hydroxylation is predicted to occur more likely in the geranyl tail, although multiple isoforms are predicted. Interestingly, an epoxide structure is also predicted, which is not an unexpected metabolic result of cytochrome P450 oxidation reactions; epoxides are often toxic due to their tendency for alkylating reactions.^86^ As such, it is crucial to differentiate between hydroxylation and epoxidation.

The in silico metabolization tools predict that the sulfonation giving rise to the *m*/*z* 555 anion is more likely to occur on the sinapoyl malate moiety of MA than on its carboxylic acid OH. Less consistency between in silico tools is observed for the site of glucuronidation of the metabolite MD: while SOMP prefers the acid to undergo glucuronidation, GLORYx predicts the hydroxyl group of sinapoyl malate as the site for glucuronidation. Further structural elucidation is therefore required for a toxicity evaluation of these metabolites. Therefore, we fractionated all metabolites from both cell lines to elucidate their structure with IRIS, as specified in Table 1.

### 3.3 Structural Elucidation of Metabolite MA

**Figure 6:**
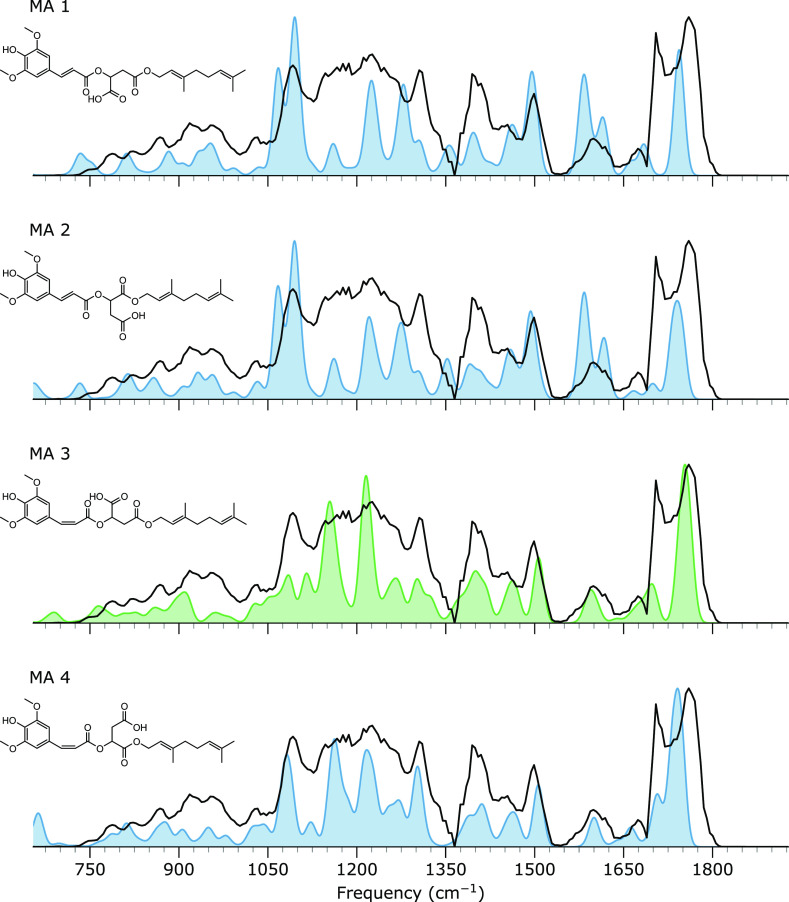
Metabolite MA. Measured IRIS spectrum of the *m*/*z* 499 ion (black trace in all panels) and computed IR spectra of different potential structures resulting from ester cleavage of DGSM, displayed as a blue or green filled curve.

Based on the mass difference between the *m*/*z* 499 ion and DGSM+Na^+^ (*m*/*z* 635), the *m*/*z* 499 ion is likely formed by ester cleavage of either of the two geraniol ester moieties. To assess this hypothesis, we recorded the IRIS spectrum of the fractioned *m*/*z* 499 ion and computed the vibrational spectra for several potential structures of this ester cleavage product. Figure 6 shows that the overall spectral matching between the experimental and computed spectra is better for the *cis*-isomerized compounds (Figure 6 MA3 and MA4). This is mainly due to two regions in the spectrum: the C=O stretching region between 1700 and 1800 cm^–1^ and the CH bending region of the alkenes between 1050 and 1125 cm^–1^, which differentiates between *cis* and *trans* CH geometries at the C=C double bond. At 1100 cm^–1^ in the predicted spectrum of Figure 6 MA3, we assign features due to CH_2_ wagging and CH bending motions of the carbons sandwiched between the two ester and carboxylic acid functional groups combined with an OH bending motion of the carboxylic acid. In contrast, in the *trans* isomers, similar vibrations combine differently, resulting in a distinct doublet rather than a singlet at around 1100 cm^–1^. As this doublet is absent in the measured spectrum, we can exclude both *trans* isomers from the annotation for the ion of interest.

The C=O stretching region is a particularly valuable structure diagnostic due to the absence of other vibrational bands and the sensitivity of the C=O stretching frequency to the chemical environment. In this range, the predicted spectrum of Figure 6 MA3 matches best with the experimental spectrum. Adopting this assignment, the peak at 1700 cm^–1^ corresponds to the C=O stretch of the geraniol ester moiety coordinating with the sodium, and the peak at 1775 cm^–1^ is due to the combined C=O stretches of the ester cleaved acid coordinating with the sodium ion and the sinapic acid ester moiety, which does not coordinate with the sodium ion. A contribution of MA4 cannot be excluded as its C=O stretch bands may be incorporated within the experimental spectral envelope. Nevertheless, we are confident that the predominantly observed ion has structure MA3.

### 3.4 Structural Elucidation of Metabolite MB

**Figure 7:**
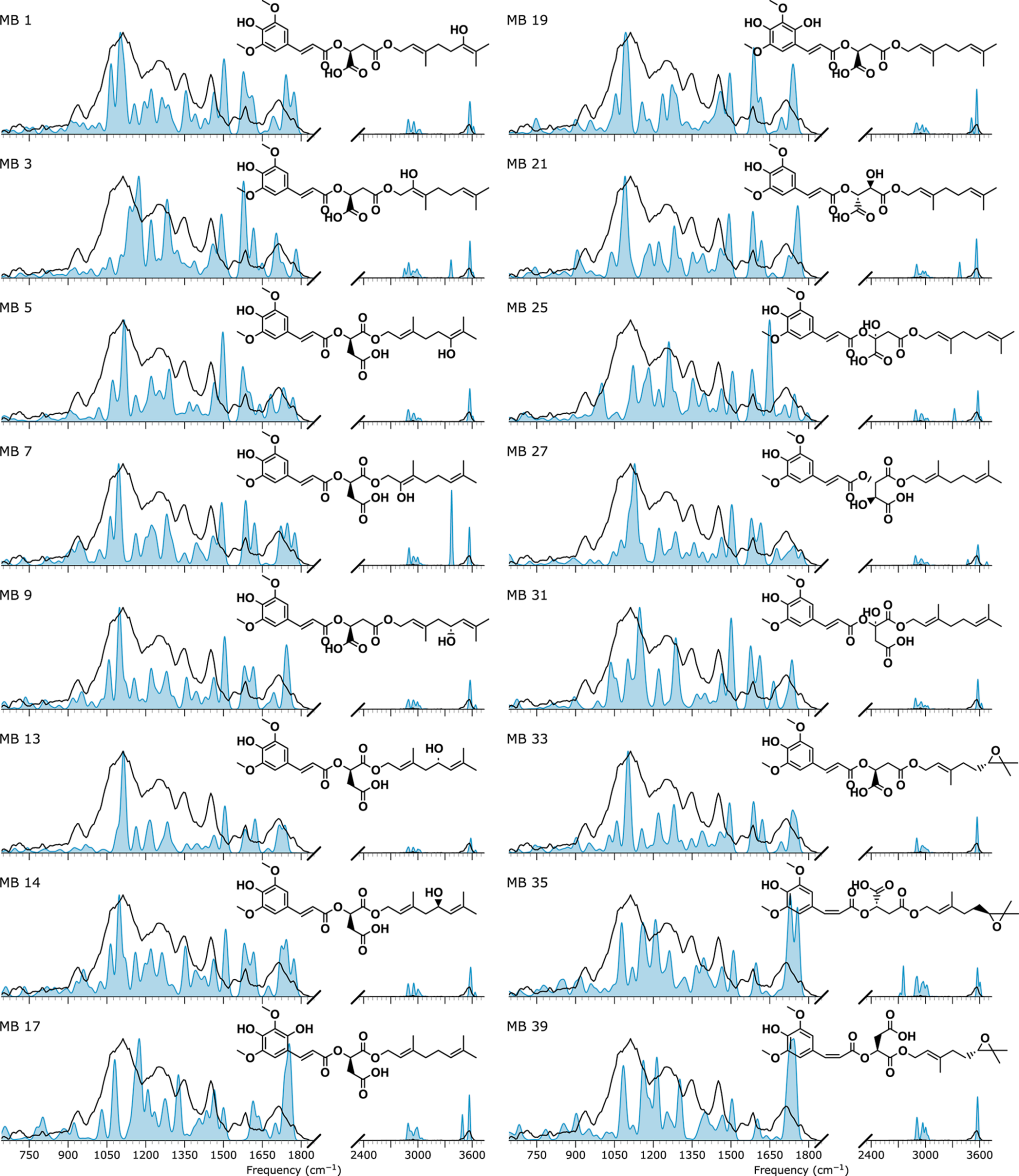
Metabolite MB. The measured IRIS spectrum of the *m*/*z* 515 ion is depicted in black, with the computed spectrum of oxidized ester-cleaved fragments of DGSM given as a blue-filled curve.

Metabolite MB, observed at *m*/*z* 515, is likely a hydroxylated product of the metabolite MA. Figure 7 compares its experimental IRIS spectrum with computed IR spectra for a subset of potential structures for MB, allowing us to eliminate several potential structures. It is challenging to assign a single isomer due to the similarity of several of the computed reference spectra and the sheer number of computed spectra. Therefore, we also examine one of the MS/MS fragment ions of MB to reduce the size of the ion and potentially simplify its IR spectrum.^87,88^ When we examine the MS/MS spectrum in Figure 4B, we observe an ion at *m*/*z* 309. This indicates a fragment with the chemical formula C_14_H_22_O_6_ (+Na^+^), which fits with an oxidation of the geraniol tail. The in silico tools (SOMP and GLORYx) indeed predict that hydroxylation will likely occur on the geraniol moieties.

**Figure 8:**
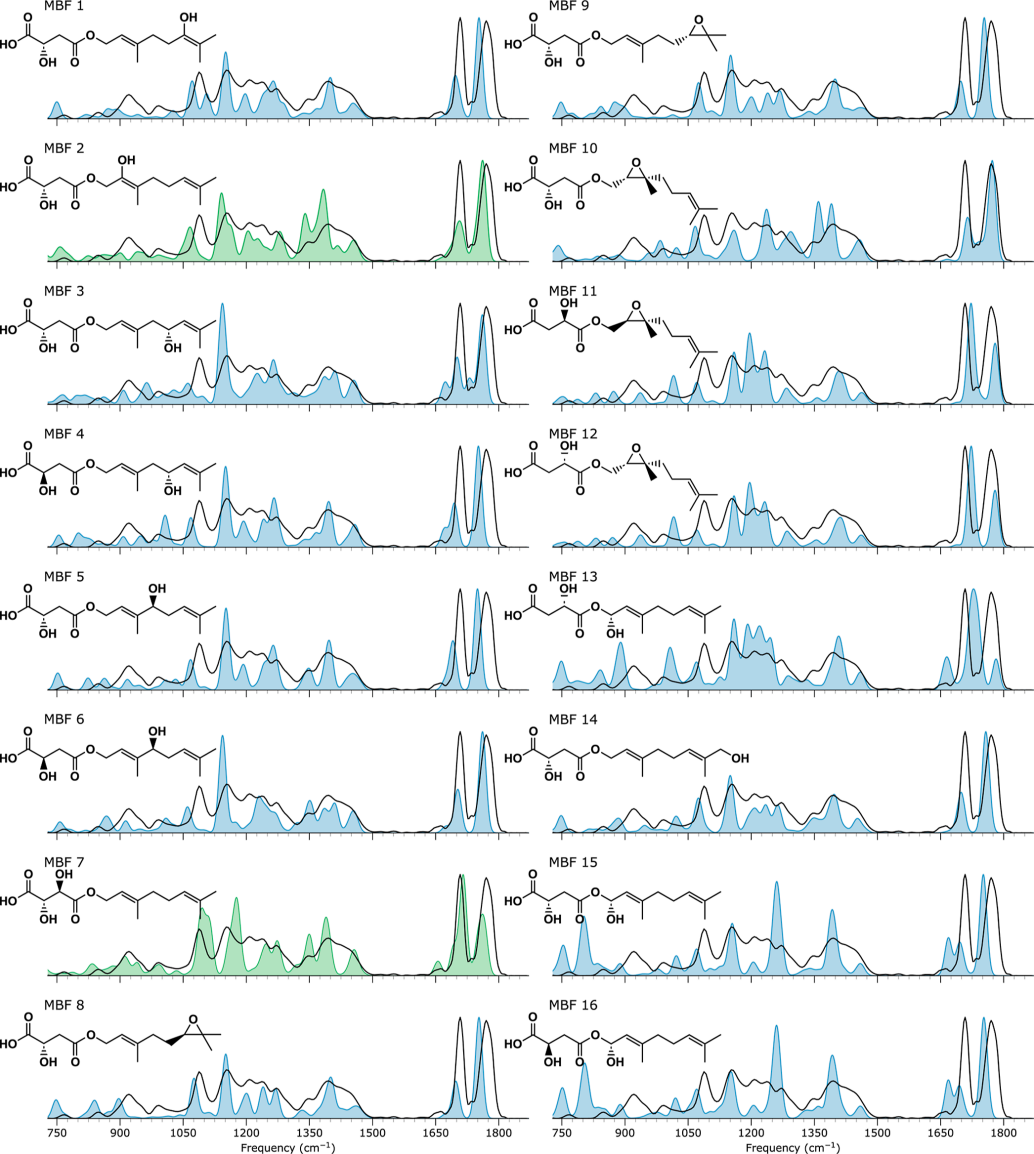
Measured IRIS spectrum of the *m*/*z* 309 CID fragment ion of metabolite MB (*m*/*z* 515) depicted in black with computed spectra of candidate structures given as blue or green filled curves.

A subset of computed IR spectra for possible structures of the *m*/*z* 309 MS/MS fragment ion is shown in Figure 8. The spectra were selected based on the presence of a doublet spectral shape in the range of carboxylic stretches at 1700 and 1770 cm^–1^. In comparing the different spectra, we note that all have a reasonable qualitative fit between 750 and 1500 cm^–1^, which does not allow us to reject structures for assignment. However, there is a minor spectral shoulder to the carboxylic stretch vibrations at 1650 cm^–1^, which is not present in all of the computed spectra. Only the predicted spectra of MBF2, 3, 7, and 10 reproduce this triplet of peaks well. Of these four spectra, MBF2 and 7 match best with the experimental spectrum below 1500 cm^–1^, with MBF7 having a slightly better match. However, when we look at the corresponding parent ions of fragments MBF2 and MBF7, respectively MB3 and MB27, in Figure 7, we see that MB3 has a predicted OH stretch that is not observed in the measured spectrum of the *m*/*z* 515 ion. Further, the qualitative match between the measured and computed spectrum of the MB27 structure is slightly better than that of MB3, though not sufficiently to prefer one over the other. Nevertheless, we assign the MB27 structure as metabolite MB, based more on the MBF7 spectrum. This tentative assignment helps us exclude the epoxide isomers as the identity of metabolite MB, which were predicted to have much higher toxicity, as further detailed below. Hence, by analyzing the IR spectra of the *m*/*z* 515 ion and its *m*/*z* 309 fragment, we can direct the in silico predictions to exclude the epoxides, even if we can only tentatively annotate the metabolite MB.

### 3.5 Structural Elucidation of Metabolite MC

**Figure 9:**
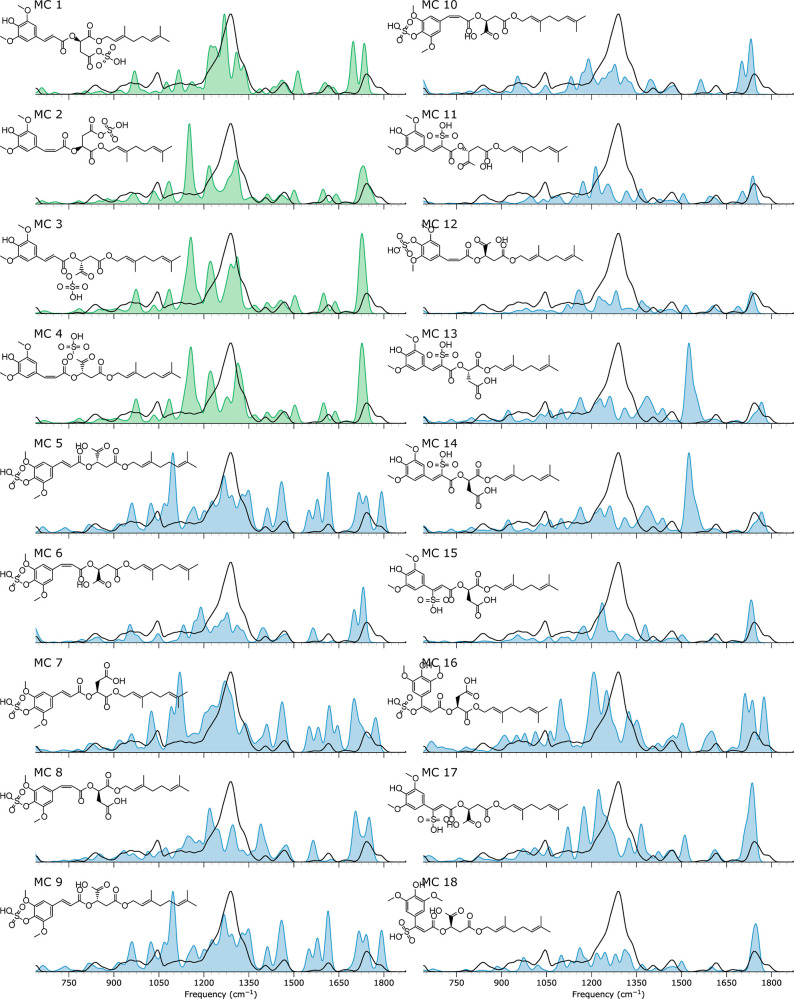
Metabolite MC. The measured IRIS spectrum of the *m*/*z* 555 ion is depicted in black, with the computed spectrum of sulfonated ester-cleaved fragments of DGSM as a blue- or green-filled curve. Note that absorption modes involving an S-atom are scaled using a 1.049 scaling factor instead of a 0.975 scaling factor.

From the HRAM mass value, the exclusive negative-ion mode observation, and the in silico predictions, we derive that the *m*/*z* 555 ion is a sulfonation product of metabolite MA. Figures 9 and S2 in the Supporting Information depict the computed IR spectra for four different sulfonation sites. Structures MC1 to MC4 result from sulfonation on the ester-cleaved acid, whereas structures MC5 to MC10 depict structures where the sinapoyl alcohol is sulfonated. MC11 to MC18 are sulfonations of the C=C double-bond carbons in either *cis* or *trans* isomerization.

Using a uniform scaling factor to correct the harmonic frequencies (Figure S2) suggests that none of the associated spectra match the measured spectrum. The main deficiency of all computed spectra is their inability to reproduce the intense absorption feature extending from 1200 to 1350 cm^–1^. Presumably, this feature corresponds to the SO stretching modes of the sulfonate moiety. Several studies have reported severe deviations in the computed harmonic frequencies of these modes using B3LYP and other DFT functionals, which can be remedied by applying a mode-specific scaling factor.^89−92^Figure 9 overlays the experimental spectrum with calculations using a scaling factor of 1.049 for the sulfonate modes instead of 0.975, which suggests that the two sulfonated acids MC1 and MC3 are promising candidates. Further support comes from the MS/MS spectrum of MC, as shown in Figure 4, where the fragment ion at *m*/*z* 223 is best explained by a deprotonated sinapic acid molecule, implying that sulfonation occurred on the geranyl tail and not on the sinapoyl moiety. However, the IR spectral match for the carboxyl stretches around 1775 cm^–1^ in MC1 and MC3 is less favorable, and MC2 and MC4 appear to be better candidates, which, on the other hand, provide a less favorable match in the sulfonate stretch region. Hence, our tentative assignment based on MS/MS and IR spectral data points toward a mixture of structures MC1 to MC4, since the quality of the IR spectral match is insufficient for a confident, unique assignment. Nevertheless, we note that this contrasts with the GLORYx prediction of sulfonation on the sinapoyl malate hydroxyl group; the mismatch of the computed spectra for those structures appears to be worse than the deficiencies for MC1–MC4.

### 3.6 Structural Elucidation of Metabolite MD

**Figure 10:**
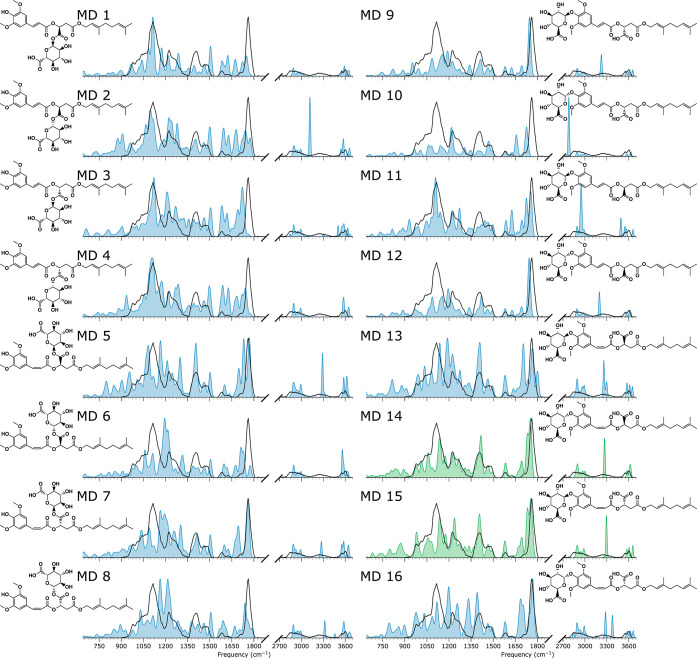
Measured IRIS spectrum of the *m*/*z* 675 ion (black trace in all panels) with the computed spectra of glucuronated ester-cleaved fragments of DGSM (blue or green filled curves). The first column (MD1–8) contains structures of the metabolite MD of Table 1 with glucuronidation on the acid of the cleaved ester moiety; the second column (MD9–16) displays structures with glucuronidation on the sinapoyl hydroxyl group.

The mass of the *m*/*z* 675 ion suggests that this feature is due to the glucuronidation of MA. The first column of Figure 10 shows structures (MD1–8) with glucuronidation on the acid of the ester-cleaved moiety of DGSM. The second column displays structures (MD9–16), where glucuronidation occurred on the hydroxyl of either of the carboxylic acids resulting from ester cleavage. The four upper rows (MD1–4 and 9–12) represent *trans*-isomers, while the lower rows (MD5–8 and 13–16) show computed spectra of *cis*-isomers. We used a process of elimination to arrive at the molecular structure. Comparing the experimental and computed spectra for the acid glucuronidation structures in their *trans*-isomerized forms (MD1–4), we observe a poor match of the C=O stretch vibration at 1750 cm^–1^, both in intensity and position. Although the *cis*-isomers MD5–8 match better in this range, the strong experimental feature around 1100 cm^–1^ is poorly reproduced for these structures, so we eliminate the *cis*-isomer forms. Hence, based on the IR spectra, glucuronidation on the carboxylic acid appears unlikely, and we turn our attention to the hydroxyl glucuronidation products in the second column. The MD9, 10, and 12 structures of Figure 10 can be safely ruled out as they fail to reproduce the strong band near 1100 cm^–1^. A mismatch in the carboxylic acid stretch in the predicted spectrum of MD11 and MD13 eliminates these structures from the remaining six candidates, especially since the relatively intense features between 1500 and 1700 cm^–1^ are absent from the experimental spectrum. The computed spectrum of MD16 predicts too many features between 1100 and 1750 cm^–1^ compared to the measured spectrum.

This leaves us with the computed spectra of MD14 and MD15, which are diastereomers and hence very similar from a molecular structure perspective. The measured signal intensity below 900 cm^–1^ is too weak to distinguish between the two structures. The calculations in the hydrogen stretch range between 2700 and 3700 cm^–1^ indicate multiple OH stretch vibrations for both structures, obviously due to the glucuronide moiety. The computed spectrum for MD14 matches the closely spaced nature of this feature slightly better than that of MD15, where the computed spectrum of MD15 predicts an OH-stretch mode at higher wavenumbers than what is observed. When we examine the OH stretches of MD14, we determine that the feature observed near 3600 cm^–1^ is due to four normal modes: one acid OH stretch and three hydroxyl OH stretches of the glucuronide. We, therefore, assign structure MD14, although we cannot exclude a mixture with MD15.

### 3.7 In Silico Toxicity

**Figure 11:**
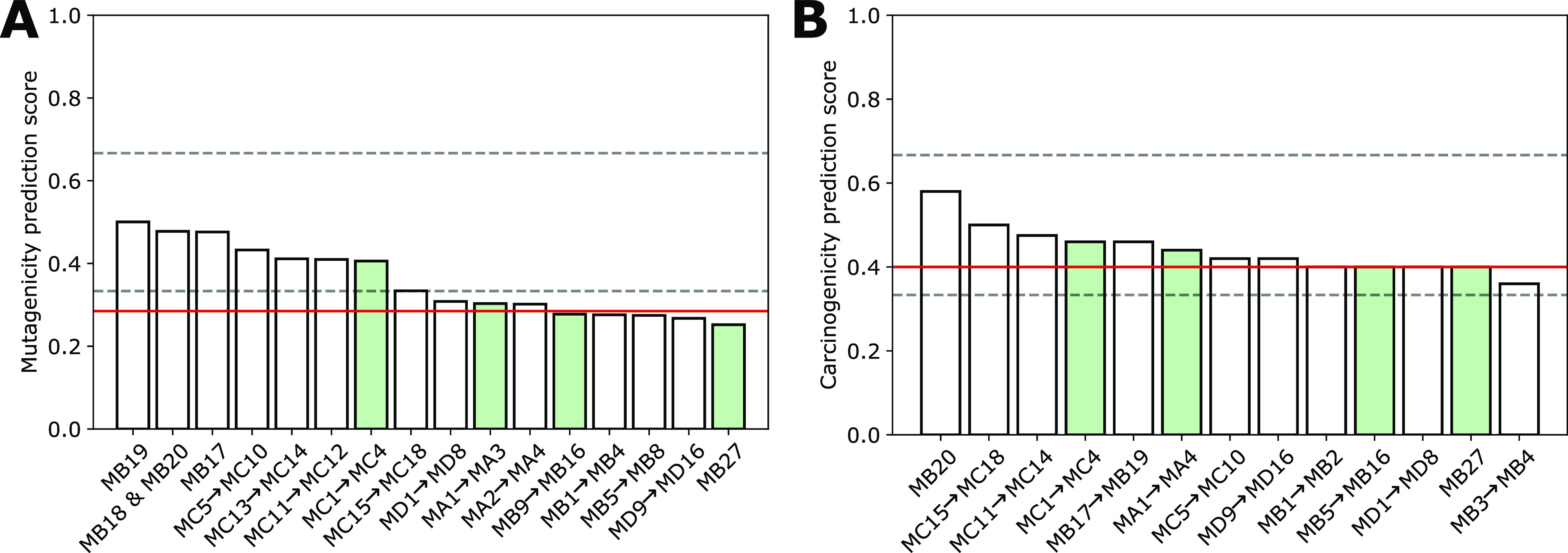
Schematic presentation of the results of the in silico analysis concerning the endpoint mutagenicity (A) and carcinogenicity (B). The test compounds are listed by their average prediction score on the *y*-axis. In addition, the prediction scores were divided into three different groups: the probable mutagens/carcinogens with scores greater than 0.66, the probably nonmutagens/noncarcinogens with scores smaller than 0.33, and the remaining equivocal predictions with scores between 0.33 and 0.66. The red line indicates the score for DGSM. Metabolites are identified with IRIS as green-filled bars (MA3, MB27, MC1–4, and MD14–15).

In order to estimate the possible toxicity of the metabolites detected following a 24 h incubation with DGSM in Caco-2 and HepaRG cells, we employed a suite of in silico models. Computations were performed for the IRIS-identified structures and the related isomers depicted in Figure 11. Sulfonated metabolites (MC1 to MC18, Figure 11A) showed mutagenicity scores ranging between the thresholds 0.5 and 0.33, indicating that they are predicted to be nonmutagenic, but the reliability is not optimal (see Method section). In contrast, the other metabolites had mutagenicity scores below 0.33, indicating that they are confidently predicted to be nonmutagenic. Carcinogenicity scores mostly ranged between the thresholds of 0.5 and 0.33. Thus, the metabolites are predicted to be noncarcinogenic, but the reliability of the predictions is again not optimal (Figure 11B). However, notably, the predictions for one of the epoxide structures, specifically the MB20 structure, have a score above 0.5 for carcinogenicity. Though this score is insufficient for a reliable carcinogenicity prediction, it would raise an alert. We can exclude this isomer based on IRIS elucidation, illustrating a case where IRIS-based structure assignment can help refine the metabolites and enable a more accurate in silico evaluation.

Overall, the scores for the produced metabolites are not significantly lower than those for DGSM (red line in the bar chart). The sulfonated metabolites even exhibit higher scores for mutagenicity. Therefore, the reliability of the predictions made for the metabolites is similar to those made for DGSM. It is important to remember that the in silico models used to make these predictions provide a qualitative estimate and do not indicate the potency (such as weak, mild, strong, very strong, etc.). As a result, it is impossible to interpret the difference in toxicity in such cases directly.

The predicted acute toxicity values ranged from 1500 to 7100 mg/kg of bw (Table 2). Notably, the hydroxylated, sulfonated, and glucuronidated metabolites had substantially higher oral LD_50_ values than the ester-cleaved metabolites, or DGSM. It is also noticeable that the position of the substituent can impact the predicted toxicity (for instance, MC15 versus MC11). This is precisely where the structural elucidation power of IRIS may aid in improving in silico toxicity assessment. When isomeric structures cannot be differentiated, it is typically assumed that the most hazardous isomer is present. However, this may only sometimes be the case; the same is true for the predicted NOAEL values. Hydroxylated, sulfonated, and glucuronidated metabolites have predicted values higher than those of DGSM or the ester-cleaved metabolites. Both the type of substitution and the substituent’s position influence the predicted toxicity, e.g., MC1 versus MC15. When we examine the NOAEL values for the epoxide isoforms of MB (MB17 → MB20), we note that the values are far lower than those of DGSM, which would raise concerns. However, as epoxidation was excluded based on IRIS characterization, we can disregard these from the NOAEL interpretation.

**Table 2:** Predicted Oral LD_50_ and NOAEL in Rats^a^

substance	LD_50_ (mg/kg bw)	NOAEL (mg/kg bw per day)
DGSM	1575	1194
**MA1** and **MA3**	**1546**	**1297**
MA2 and MA4	1574	1297
MB20	2902	216
MB19	2965	216
MB18	3053	216
MB17	3091	216
MB1 and MB2	3398	2150
MB3 and MB4	3686	2150
MB5 and MB6	3464	2150
MB7 and MB8	3874	2150
MB9 → MB12	4804	1695
**MB27**	**4866**	**4272**
MB13 → MB16	4888	1695
**MC1 → MC4**	**4789**	**3840**
MC5 → MC10	4344	3840
MC11 and MC12	4054	1441
MC13 and MC14	4054	1587
MC15 → MC18	7062	1322
MD1 → MD8	4101	5211
**MD9 → MD16**	**6416**	**6045**

a Metabolites identified with IRIS are MA3, MB27, MC1-4, and MD14-15.

Regarding endocrine toxicity, DGSM and most metabolites were predicted to have a binding affinity for the estrogen receptor, albeit with low reliability (Table 3). Some isomers, though, were predicted to have no binding affinity with moderate reliability (e.g., MB7, MB8, and MD9 → MD18), emphasizing the importance of knowing the substitution site. All metabolites and DGSM were predicted to be inactive regarding estrogen receptor-mediated effects, with good reliability, except for a few sulfonated metabolites (Table 3). Moreover, all metabolites were predicted to be inactive toward the androgen receptor, with moderate reliability for all except for MC15 → MC18, where the reliability was low. None of the test compounds were predicted to exert effects via thyroid receptors (all predictions had good reliability except a few sulfonated metabolites that had moderate reliability).

**Table 3:** Predictions of Endocrine Toxicity Using the VEGA Platform^a^

substance	ER relative binding affinity	ER-mediated effect	AR-mediated effect	TR alpha effect	TR beta effect
DGSM	active^b^	inactive^c^	inactive^c^	inactive^d^	inactive^d^
**MA1 → MA4**	**active**^b^	**inactive**^d^	**inactive**^c^	**inactive**^d^	**inactive**^d^
MB1 → MB6	active^b^	inactive^d^	inactive^c^	inactive^d^	inactive^d^
MB7 → MB8	inactive^c^	inactive^d^	inactive^c^	inactive^d^	inactive^d^
MB9 → MB16	active^b^	inactive^d^	inactive^c^	inactive^d^	inactive^d^
MB17→MB20	active^b^	inactive^d^	inactive^c^	inactive^d^	inactive^d^
**MB27**	**active**^b^	**inactive**^d^	**inactive**^c^	**inactive**^d^	**inactive**^d^
**MC1 → MC4**	**active**^b^	**inactive**^d^	**inactive**^c^	**inactive**^d^	**inactive**^d^
MC5 → MC14	inactive^b^	inactive^d^	inactive^c^	inactive^d^	inactive^d^
MC15 → MC18	inactive^b^	inactive^b^	inactive^b^	inactive^c^	inactive^c^
MD1 → MD8	active^b^	inactive^d^	inactive^c^	inactive^d^	inactive^d^
**MD9 → MD16**	**inactive**^c^	**inactive**^d^	**inactive**^c^	**inactive**^d^	**inactive**^d^

a Metabolites identified with IRIS are MA3, MB27, MC1-4, and MD14-15.

b Low reliability.

c Moderate reliability.

d Good reliability.

## 4 Conclusions

This study demonstrates the effectiveness of LC–MS–IRIS identification in identifying the chemical structure of metabolic transformation products, even without the aid of reference compounds, by utilizing DFT-computed IR spectra. A precise structural assignment using IRIS can remain challenging for large “small molecules”, such as the DGSM metabolites studied here. This is partially due to the extensive conformational freedom of the geraniol tails, challenging our conformational search workflow and the related likeliness of finding many low-energy conformers. The Boltzmann-weighted mixing of computed spectra depends strongly on the accuracy of the relative energies computed, which is estimated to be on the order of several kJ/mol, at best. Nonetheless, we have shown that LC–MS–IRIS elucidation can confidently exclude many isomers from assignment, thereby improving the subsequent interpretation of in silico toxicity predictions.

Our study indicates that the elucidated metabolic transformation products of DGSM are unlikely to pose a significant toxicity risk. This is also indicated by the lack of cytotoxicity in cell assays of Caco-2 and HepaRG after 24 h with DGSM at concentrations of up to 5 mM. Although the examined agrochemical compound did not raise any toxicity alerts in this case, IRIS may, in other cases, be used to delineate structures of metabolic products so that in silico toxicity screening can be performed with more stringent boundary conditions.

## Supporting Information Available

Negative LC analysis of the cell assay samples, overview of the SOMP and GLORYx outputs, and computed spectra for the sulfonated metabolite using a uniform scaling factor of 0.975 for all vibrational modes (PDF)
