# Supplementary material for: Structural Elucidation of Agrochemical Metabolic Transformation Products Based on Infrared Ion Spectroscopy to Improve In Silico Toxicity Assessment
Source: Chem Res Toxicol. 2023 Dec 20;37(1):81–97. doi: 10.1021/acs.chemrestox.3c00316 (PMC10792670; doi:10.1021/acs.chemrestox.3c00316)
Supplement: Supplementary file 1 — tx3c00316_si_001.pdf [file tx3c00316_si_001.pdf]

Supporting Information

Structural elucidation of agrochemical metabolic transformation products based on infrared ion spectroscopy to improve in silico toxicity assessment

Matthias J.A. Vink[†], Jimmy Alarcan[‡], Jonathan Martens[†], Wybren Jan Buma[†,§], Albert Braeuning[‡], Giel Berden[†*], Jos Oomens[†,§*]

[†]*Radboud University, Institute for Molecules and Materials, FELIX Laboratory, Toernooiveld 7, 6525 ED Nijmegen, The Netherlands*

[‡]*German Federal Institute for Risk Assessment, Department of Food Safety, Max-Dohrn-Str. 8-10 10589 Berlin, Germany*

[§]*University of Amsterdam, van 't Hoff Institute for Molecular Sciences, Science Park 904, 1098 XH Amsterdam, The Netherlands*

*Corresponding authors: giel.berden@ru.nl, jos.oomens@ru.nl

This Supporting Information includes 6 pages, 2 figures and 2 tables.

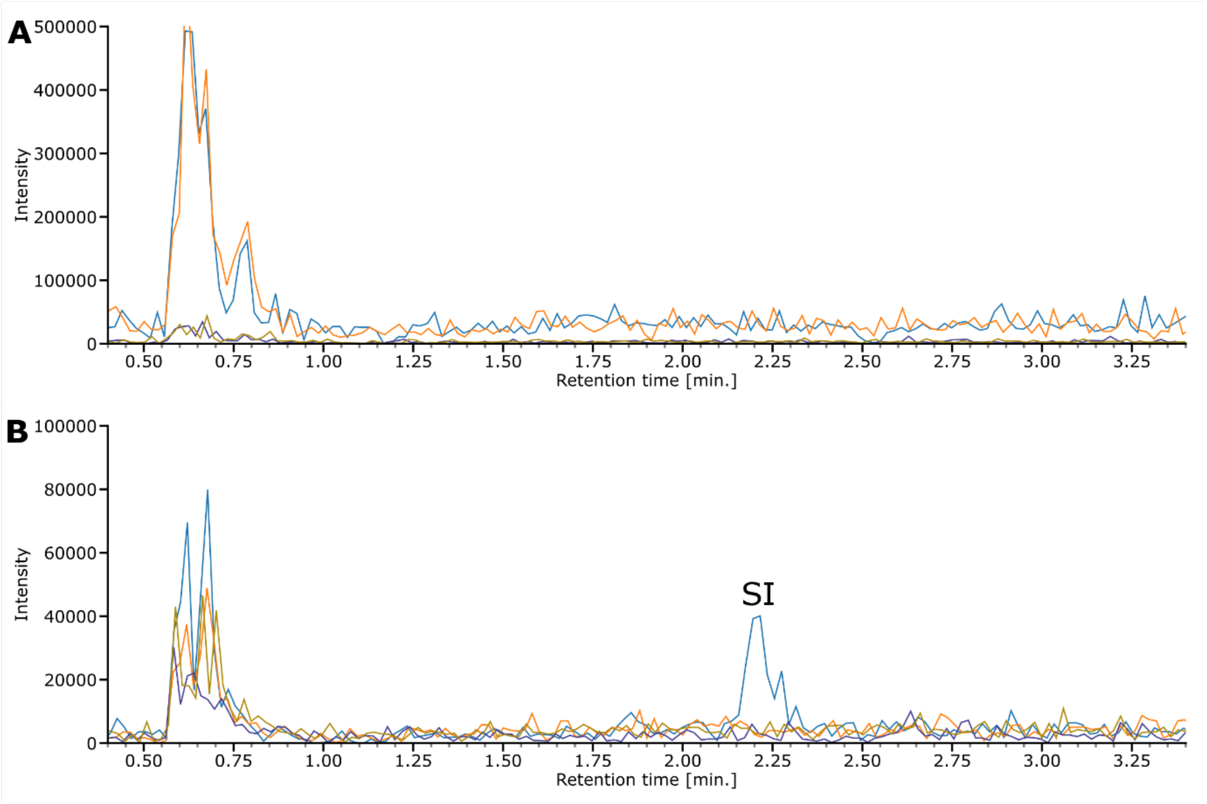

Figure S1: *m/z 555 in negative ion mode EIC curves of the LC-MS analysis of cell line assays.* ***A:*** *Caco-2 cell line assay extracts,* ***B:*** *HepaRG cell line assay extracts. In both panels, the blue trace represents incubation with DGSM, the purple curve represents DGSM incubation without cells being present in the medium, the orange trace corresponds to cells incubated with DMSO, where the brown curve represents DMSO incubation without cells present in the medium.*

Table S1: *Predicted metabolites using the GLORYx tool (input structure MA3)*

Substance	Formula	Structure
MA3	$C_{25}H_{32}O_9$	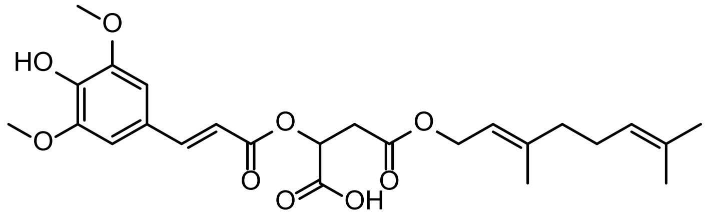
#1 (0.88)	$C_{25}H_{32}O_{12}S$	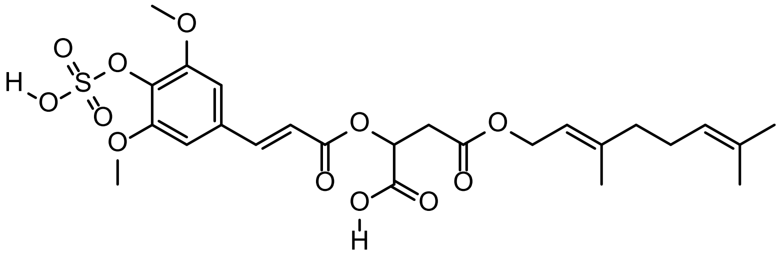
#2 (0.50)	$C_{31}H_{40}O_{15}$	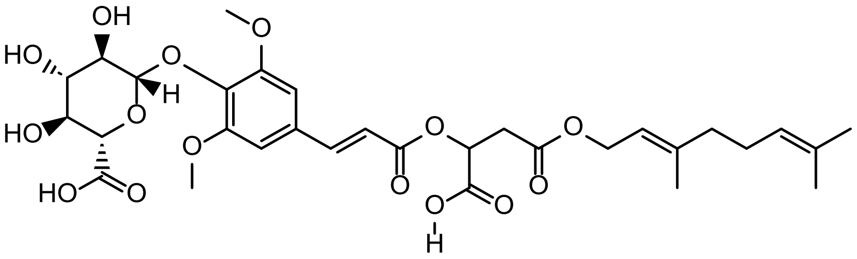
#3 (0.38)	$C_{31}H_{40}O_{15}$	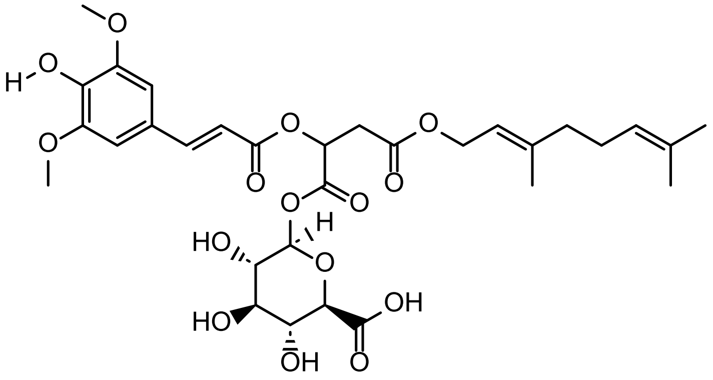
#4 (0.28)	$C_{25}H_{30}O_9$	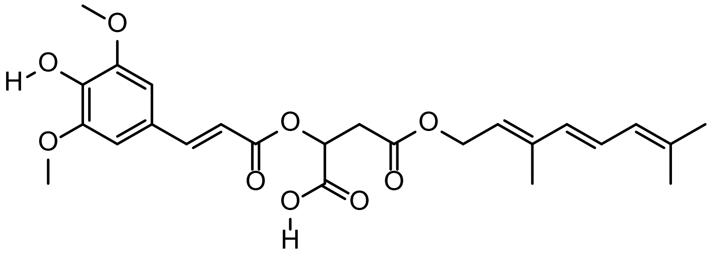

#5 (0.28) $C_{25}H_{32}O_{10}$

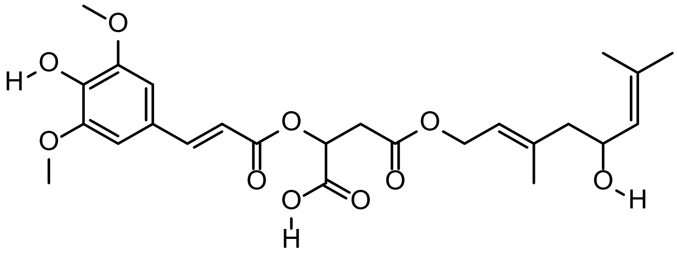

#6 (0.28) $C_{25}H_{32}O_{10}$

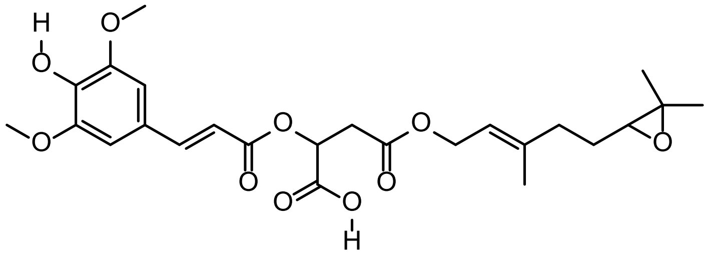

#7 (0.26) $C_{25}H_{32}O_{10}$

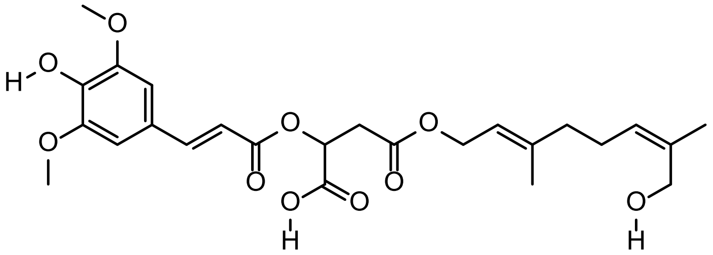

Table S2: *Predicted reactivity of UGT (Uridine 5'-diphospho-glucuronosyltransferase) per position on DGSM using the SOMP tool. Higher Delta P indicates a higher probability for glucuronidation.*

Rang	Atom number	Delta P	Rang	Atom number	Delta P
1	12	0.255	18	33	-0.194
2	34	0.214	19	17	-0.206
3	16	0.056	20	24	-0.215
4	9	-0.014	21	8	-0.222
5	27	-0.028	22	10	-0.225
6	13	-0.046	23	5	-0.275
7	2	-0.048	24	18	-0.302
8	28	-0.048	25	23	-0.312
9	31	-0.048	26	3	-0.331
10	1	-0.135	27	30	-0.331
11	32	-0.135	28	21	-0.337
12	11	-0.152	29	4	-0.34
13	20	-0.159	30	29	-0.34
14	14	-0.163	31	19	-0.345
15	15	-0.169	32	7	-0.362
16	25	-0.178	33	22	-0.376
17	26	-0.178	34	6	-0.396

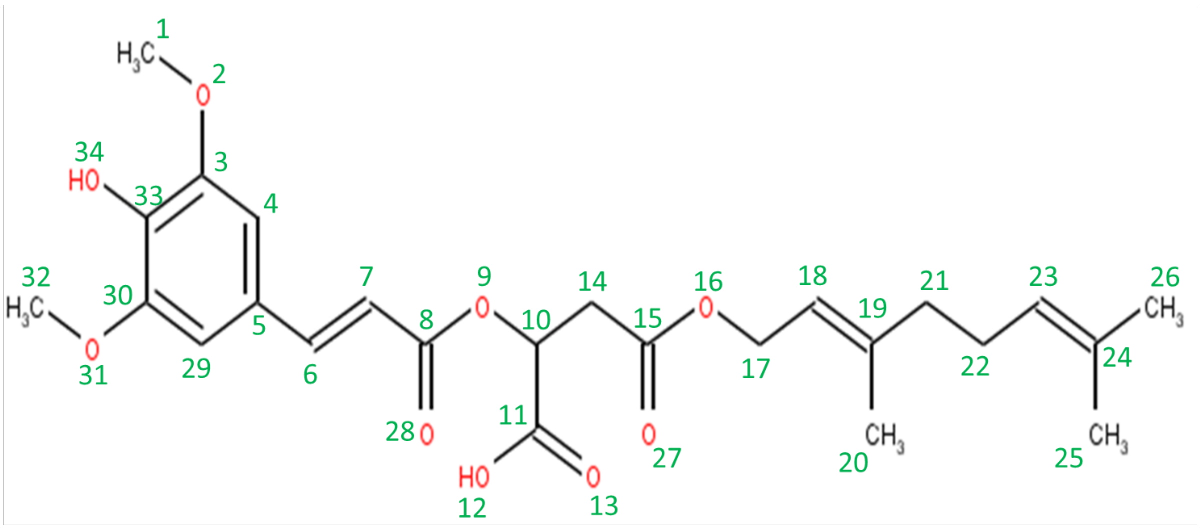

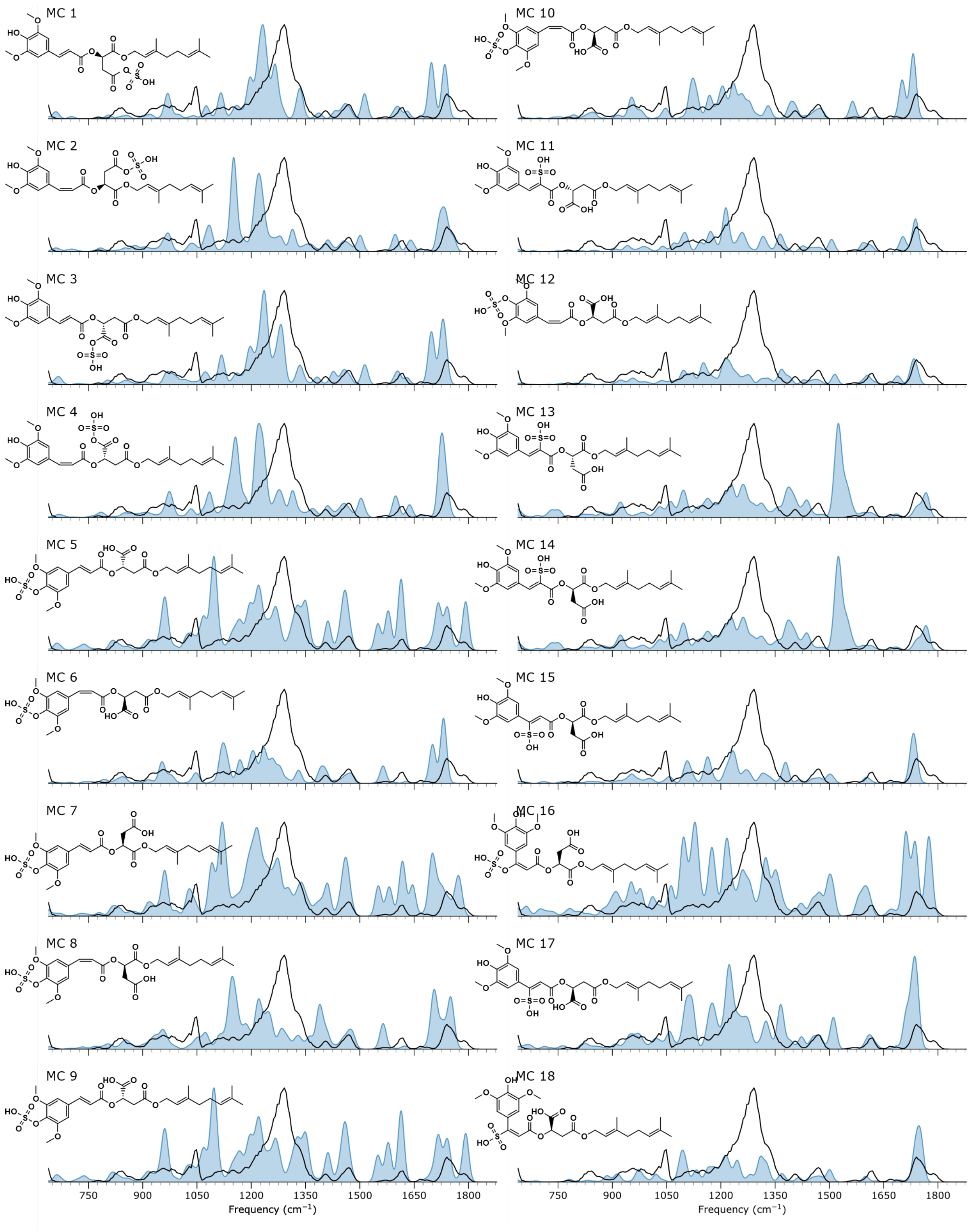

Figure S2: *Metabolite MC. Measured IRIS spectrum of the m/z 555 ion depicted in black with the computed spectra of sulfonated ester-cleaved fragments of DGSM in different isomeric forms given as a blue filled curve. Harmonic frequencies are scaled uniformly by a factor of 0.975.*
